# miR-1236-3p targets Toll-like receptor 4 to suppress the anti-*Mycobacterium tuberculosis* activity of macrophage

**DOI:** 10.1016/j.isci.2025.112522

**Published:** 2025-05-08

**Authors:** Yating Zhang, Jie Han, Qianwei Yang, Xiaogang Cui, Huiping Duan, Ting Wu, Changxin Wu, Li Xing, Qunqun Liu, Li Dong

**Affiliations:** 1 Institutes of Biomedical Sciences, Shanxi University, 92 Wucheng Road, Taiyuan, Shanxi Province 030006, China; 2 The Fourth People’s Hospital of Taiyuan, 231 Xikuang Street, Taiyuan 030006, China; 3 Shanxi Provincial Key Laboratory of Medical Molecular Cell Biology, Shanxi University, 92 Wucheng Road, Taiyuan 030006, China; 4 Key Laboratory of Chemical Biology and Molecular Engineering of Ministry of Education, Shanxi University, Taiyuan 030006, China

**Keywords:** Immunology, Immune response, Microbiology

## Abstract

*Mycobacterium tuberculosis* (*Mtb*) modulates host innate immunity via Toll-like receptor 4 (TLR4), associated with the susceptibility to *Mtb*. Bioinformatics predicted miR-1236-3p could be a potential target for the 3′-UTR of the *TLR4* gene. However, the clinical significance and underlying mechanisms remain unclear. To validate this, we analyzed miR-1236-3p levels in 81 subjects and observed that both active tuberculosis (ATB) and latent tuberculosis infection (LTBI) patients exhibited elevated miR-1236-3p levels compared to healthy control (HC) subjects. *In vitro* dual-luciferase reporter assays confirmed that miR-1236-3p specifically targeted the 3′-UTR of *TLR4* mRNA. During *Mtb* infection in macrophages, miR-1236-3p enhanced the NF-κB signaling and reduced the release of intracellular inflammatory factors, reactive oxygen species, and nitric oxide (NO), indicating that the ability of macrophages to constrain intracellular *Mtb* infection was compromised by miR-1236-3p. In summary, miR-1236-3p may target *TLR4*/NF-κB signaling to suppress the intrinsic anti-*Mtb* activity of macrophage.

## Highlights

• Elevated miR-1236-3p and reduced TLR4 levels were observed in ATB/LTBI compared to HC
• miR-1236-3p binds to 3′UTR of TLR4 (rs11536889), thereby suppressing its translation
• TLR4 downregulation inhibits ROS/NO and NF-κB pathways, thus promoting the survival of *Mtb*

## Graphical abstract

**Figure:**
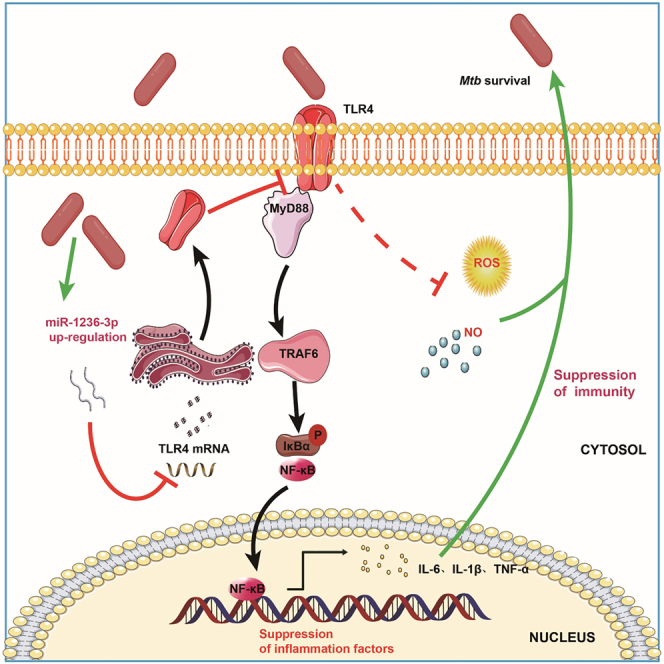

## Introduction

Tuberculosis (TB), caused by *Mycobacterium tuberculosis* (*Mtb*), remains a serious global health threat.^1^ During human infection, *Mtb* was usually inhaled into the lower respiratory tract and then recognized by alveolar macrophages, where membrane Toll-like receptors (TLRs) are involved in the process of phagocytosis and pinocytosis of *Mtb* and spark the innate immune response.^2^ In-depth understanding of the intimate interaction between *Mtb* and host immune system is required for the development of novel therapeutics.

*Mtb* can modulate the function of macrophage to escape the immune response by inhibiting the production of pro-inflammatory cytokines,^2^ neutralizing acid environment,^3^ curbing the formation of apoptotic envelope,^4^ and modifying microRNA (miRNAs) profiles.^5^

miRNA is a class of small non-coding RNA that can target specific mRNAs by base-pairing to inhibit protein translation.^6^ Single nucleotide polymorphism (SNP) in target regions of mRNAs could alter miRNA-mRNA binding affinity or specificity.^7^^,^^8^ miR-1236-3p was reported to inhibit the invasion and proliferation of *Helicobacter pylori*-related gastric cancer cells by downregulating the expression of metastasis-associated protein 2 (MTA2).^9^ Overexpression of X protein of hepatitis B virus resulted in the significant decrement of miR-1236-3p and increment of alpha-fetoprotein (AFP) in the hepatoma cells.^10^ The miR-1236-3p has been also implicated in the pathogenesis of both liver cancer and renal cell carcinoma.^11^^,^^12^ While we previously found that the Toll-like receptor 4 (TLR4) polymorphism c.1205G (rs11536889) was associated with the susceptibility to TB,^13^ but the underlying mechanism is unknown. Bioinformatic analysis revealed that miR-1236-3p may target the wild-type c.1205G (rs11536889), rather than the mutant type c.1205C of *TLR4*. However, the clinical significance and role of miR-1236-3p in the pathogenesis of TB have yet to be investigated.

In this study, we analyzed the clinical samples and performed *in vitro* experiments to explore the role of miR-1236-3p in *Mtb* infection and found that *Mtb* infection could increase miR-1236-3p to suppress TLR4/NF-κB signaling, leading to compromised activity of macrophages and impaired control of *Mtb* infection.

## Results

### Sociodemographic characteristics of subjects

A total of 81 subjects were recruited from the Chinese Han population, including 27 active pulmonary tuberculosis (ATB) patients, 28 latent tuberculosis infection (LTBI) patients, and 26 health controls (HCs). The sociodemographic characteristics of these subjects were shown in Table 1.

**Table 1:** Sociodemographic characteristics of 27 ATB patients, 28 LTBI patients, and 26 HC subjects

Characteristics	Total^b^^,^ n (%)	ATB patients n (%)	LTBI patients n (%)	HC subjects n (%)	*p*-Value^c^	ATB patients/HC^d^ subjects		LTBI patients/HC^d^ subjects	
						OR (95% CI)	*p*-Value	OR (95% CI)	*p*-Value
Total	81	27	28	26					
Age, years^a^	36 ± 12	36 ± 18	34 ± 8	39 ± 8	0.289				
Gender					0.470				
Male	39(48.2)	15 (55.6)	11(39.3)	13 (50.0)					
Female	42(51.8)	12(44.4)	17(60.7)	13(50.0)					
Smoking					0.822				
Yes	18(22.2)	5(18.5)	6(21.4)	7(26.9)					
No	61(75.3)	20(74.1)	22(78.6)	19(73.1)					
Drinking					0.006	0.436 (0.062–3.087)	0.406	0.132 (0.007–0.574)	0.007
Yes	25(30.9)	7(25.9)	4(14.3)	14(53.9)					
No	51(63.0)	17(63.0)	23(82.1)	11(42.3)					
Education level					**<** 0.001	NA	NA	NA	NA
Junior school or lower	7(8.6)	7(25.9)	0(0.0)	0(0.0)					
Senior high school and higher	73(90.1)	19(70.4)	26(92.9)	26(100.0)					
Marital Status					0.009	9.600 (1.881–48.999)	0.007	0.318 (0.347–3.467)	0.347
Married	55(67.9)	15(44.4)	21(75.0)	24(92.3)					
Unmarried/Divorced/Widowed	24(29.6)	12 (55.6)	7(25.0)	2(7.7)					
History of infectious disease					0.008	11.818 (0.563–247.971)	0.112	NA	NA
Yes	7(8.6)	6(22.2)	0(0.0)	1(3.9)					
No	74(91.4)	21(77.8)	28(100.0)	25(96.1)					
miRNA^e^						10.102 (1.550-65.847)	0.016	5.675 (0.025-25.878)	0.025
TLR4^f^						0.077 (0.007-0.867)	0.038	0.067 (0.010-0.435)	0.005

a Differences in mean age between ATB patients, LTBI patients and HC subjects were analyzed using an unpaired t-test.

b Due to incomplete information collection, the actual sample size was not 81 for certain items.

c Comparisons of age, sex, smoking, drinking, education level, and marital status between ATB, LTBI, and healthy controls were performed by using Pearson’s Chi-Squared test.

d *p*-values, odds ratios (ORs) and 95% confidence intervals (CIs) were calculated by logistic regression. In the independent variables of marital status, education level, drinking, and history of infectious disease, the subjects with married status, junior school or lower education, no alcohol, and no history of infectious disease are used as controls.

e ATB miR-1236-3p, with 3/4 of HC miR-1236-3p as the cut-off value; LTBI miRNA, with 1/2 of HC miR-1236-3p as the cut-off value.

f ATB TLR4, with 1/2 of HC TLR4 as the cut-off value; LTBI TLR4, with 1/2 of HC TLR4 as the cut-off value. ATB, Active tuberculosis; LTBI, Latent tuberculosis infection ; HC, Healthy control; SD, Standard deviation; NA, non-applicable.

### *Mtb* infection increases the level of miR-1236-3p

**Figure 1:**
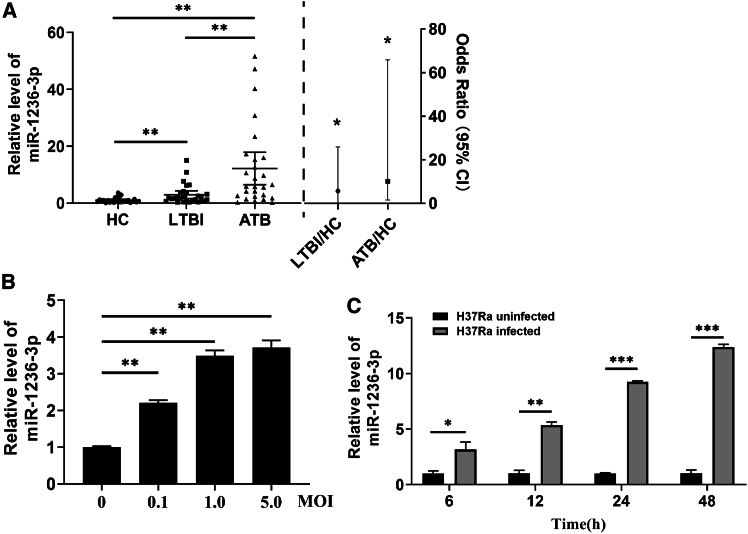
The higher miR-1236-3p levels in the human peripheral blood of TB patients and THP-1 cells infected with *Mtb* H37Ra (A) miR-1236-3p levels increased in ATB patients (*n* = 27) and LTBI patients (*n* = 28) compared with those in HC subjects (*n* = 26). The miR-1236-3p levels of LTBI and TB patients were compared to those of HC subjects by logistic regression calculation. (B) miR-1236-3p levels were increased by *Mtb* H37Ra infection in THP-1-derived macrophages. Cells were infected with H37Ra with MOI at 0.1 to 5.0 and miR-1236-3p level was determined at 24h postinfection. (C) miR-1236-3p levels were increased in THP-1 cells by H37Ra infection at 5.0 MOI. All data are presented as mean ± SD from three independent experiments. Student’s *t* test was used for two groups and one-way ANOVA was used for the comparison of more than two groups. ∗, *p* < 0.05; ∗∗, *p* < 0.01; ∗∗∗, *p* < 0.001.

The level of miR-1236-3p in the peripheral blood cells of subjects was determined using quantitative RT-PCR (RT-qPCR), in which the intracellular small nuclear RNA (snRNA) U6 transcript was used as an internal reference. miR-1236-3p levels were significantly higher in ATB (OR = 10.1020, *p* = 0.016) and LTBI (OR = 5.675, *p* = 0.025) than in HC (Figure 1A, Table 1). Logistic regression calculation also showed the significant upregulation of miR-1236-3p in LTBI and ATB patints compared to HC (Table 1).

To further explore the correlation of miR-1236-3p with *Mtb* infection, we performed experiments using human THP-1-derived macrophages. THP-1 cell was differentiated into a macrophage by PMA treatment for 24h, and then infected with *Mtb* H37Ra for 24 h with multiplicity of infection (MOI) at 0.1, 1.0, and 5.0. The level of miR-1236-3p was determined by RT-qPCR at 24h post-infection. H37Ra infection significantly increased the miR-1236-3p level (Figure 1B). Next, THP-1-derived macrophages were infected with H37Ra at 5.0 MOI, and then the level of miR-1236-3p was determined at 6, 12, 24, and 48h post-infection. MiR-1236-3p level was increased over the time course of infection in macrophages (Figure 1C). The results indicated that *Mtb* infection increased the expression of miR-1236-3p in macrophages, which mechanistically contributed to the high level of miR-1236-3p in ATB and LTBI (Figure 1A).

### miR-1236-3p inhibits the translation of TLR4 in macrophages

**Figure 2:**
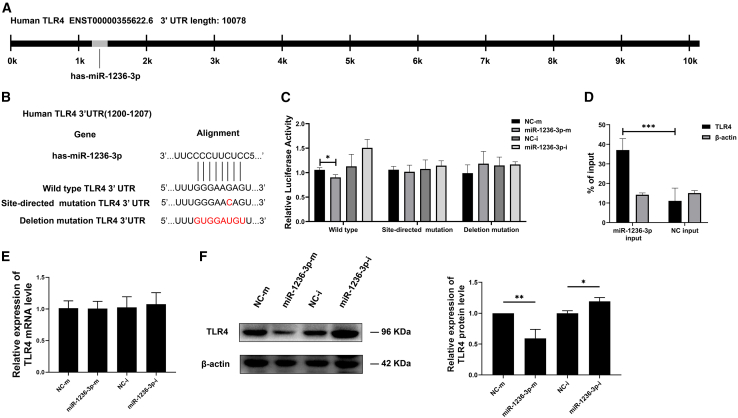
miR-1236-3p targets the 3′-UTR of TLR4 mRNA and inhibits TLR4 at the translational level (A and B) Schematic representation of the TLR4 3′-UTR showing the putative binding site and the mutation site for miR-1236-3p. (C) Luciferase reporter assay to verify the effect of miRNA mimic and inhibitor in THP-1 cells. Luciferase activity was measured in THP-1 cells transfected with wild type (WT) vector or mutant TLR4 vector together with miR-1236-3p mimic or inhibitor. (D) miR-1236-3p pulldown enrichment effect was measured by performing Biotinylated Micro-RNA Pull Down Assay. (E) RT-qPCR to determine TLR4 mRNA levels in THP-1 cells transfected with miR-1236-3p mimic or inhibitor. (F) TLR4 protein levels in THP-1 cells transfected with miR-1236-3p mimic or inhibitor. TLR4 protein level was analyzed using Western blotting. All data are presented as mean ± SD from three independent experiments. Student’s *t* test was used for two groups and one-way ANOVA was used for the comparison of more than two groups. ∗, *p* < 0.05; ∗∗, *p* < 0.01; ∗∗∗, *p* < 0.001.

To identify the target of miR-1236-3p, we first utilized TargetScan Release 8.0 platform^14^ and found predicted that miR-1236-3p could bind to the wild-type c.1205G (rs11536889) 3′-UTR of *TLR4* (Figures 2A and 2B). TLR4 plays a crucial role in both innate and adaptive immune responses to *Mtb* infection.^15^ To verify this miRNA binding site, the potential binding sequence in the TLR4 3′-UTR was inserted into 3′-UTR of luciferase reporter gene in psiCHECK-2 vector to produce wild-type vector. The potential miRNA binding site in wild-type vector was then site-directed or deletion mutated to produce mutant vectors that were used as negative controls (NC) (Figure 2B). Those vectors were co-transfected into THP-1-derived macrophages together with miR-1236-3p mimic or the inhibitor, and the luciferase activity was determined at 48h post-transfection. As shown in Figure 2C, the presence of miR-1236-3p mimics repressed the activity of luciferase in wild-type plasmid-transfected cells, but had no inhibitory effects on the relative luciferase activity of luciferase reporter plasmids containing single-point mutation or deletion mutation. In addition, a miRNA pulldown experiment was performed to further verify the binding of miR-1236-3p to TLR4 sequences. The results showed that miR-1236-3p can successfully pull down the TLR4 specific sequences (Figure 2D). The results demonstrated that rs11536889 3′-UTR of TLR4 was targeted by miR-1236-3p.

The effects of miR-1236-3p mimic and inhibitor on the level of endogenous TLR4 mRNA or protein were examined in THP-1-derived macrophages. As shown in Figure 2E, no obvious change was detected in TLR4 transcript level after transfection with miR-1236-3p mimic or inhibitor, but TLR4 protein was significantly decreased in miR-1236-3p mimic group and significantly elevated in the miR-1236-3p inhibitor group (Figure 2F). Taken together, these data indicate that the 3′-UTR of TLR4 is a direct target of miR-1236-3p in macrophages, which represses the translation of TLR4.

### TLR4 increases in the early stage of *Mtb* infection, and subsequently declined

**Figure 3:**
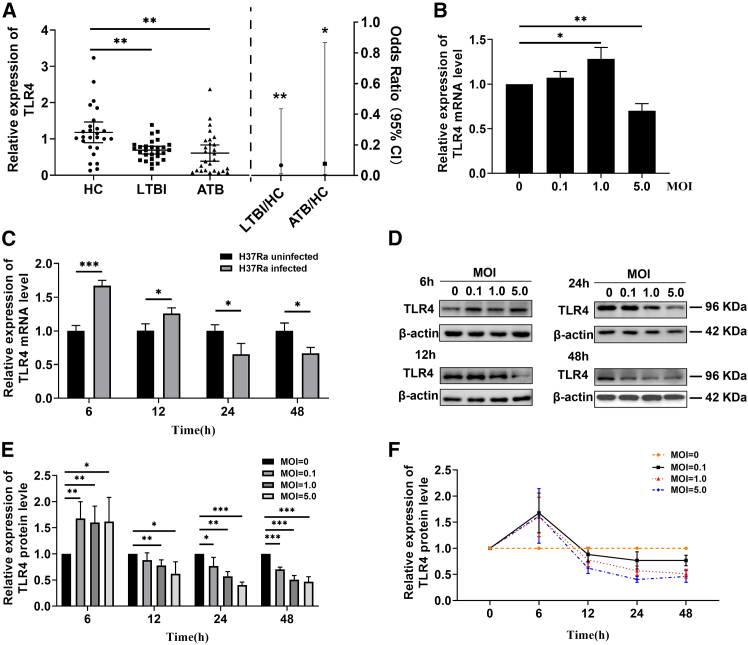
TLR4 was activated at early stage of *Mtb* infection and then significantly inhibited over the extended infection (A) TLR4 mRNA levels were lower in both ATB patients (*n* = 27) and LTBI patients (*n* = 28) compared to HC subjects (*n* = 26). The relative TLR4 mRNA level in LTBI and ATB patients was compared to that of HC subjects by logistic regression calculation. (B) The transcript level of TLR4 in THP-1-derived macrophages infected with H37Ra (MOI = 0.1, 1.0, 5.0) at 24h. (C) The transcriptional level of TLR4 in THP-1-derived macrophages infected with H37Ra at 5.0 MOI for 6h to 48h. (D and E) Western blot analysis of TLR4 protein in THP-1-derived macrophages infected with H37Ra (MOI = 0, 0.1, 1.0, 5.0) for 6h to 48h and the trendline chart analysis (F). Student’s *t* test was used for two groups and one-way ANOVA was used for the comparison of more than two groups. ∗, *p* < 0.05; ∗∗, *p* < 0.01; ∗∗∗, *p* < 0.001.

The relationship between TLR4 and TB status or bacterial burden of *Mtb* infection was explored by determining *TLR4* mRNA levels in peripheral blood of ATB patients, LTBI patients, and HC subjects (Figure 3A). *TLR4* mRNA levels were markedly lower in the ATB (*p* = 0.038) and LTBI patients (*p* = 0.005) than HC subjects (Figure 3A, Table 1). Logistic regression calculation also showed that TLR4 was significantly downregulated in LTBI and TB patients compared to HC (Table 1). The severity of tuberculosis tends to be negatively correlated with TLR4 expression level. This phenomenon was further tested using THP-1-derived macrophages infected with H37Ra, where *TLR4* transcripts significantly declined over the increasing MOI (Figure 3B) or over the increasing period of infection when cells were infected with H37Ra at the 5.0 MOI (Figure 3C). To detect the TLR4 protein, THP-1 cells were infected with H37Ra at 0, 0.1, 1.0, or 5.0 MOI respectively for 6h, 12h, 24h, or 48h, and thenwestern blot analysis was performed (Figures 3D and 3E). The results show that H37Ra infection upregulated TLR4 at early stage, but downregulated for longer infection period or at high MOI infection (Figure 3F). The results suggest that TLR4 may be temporally activated at early infection, but decreased with long-term or high burden *Mtb* infection.

### miR-1236-3p affects *Mtb* survival and regulates the production of ROS and nitric oxide (NO) by targeting TLR4

**Figure 4:**
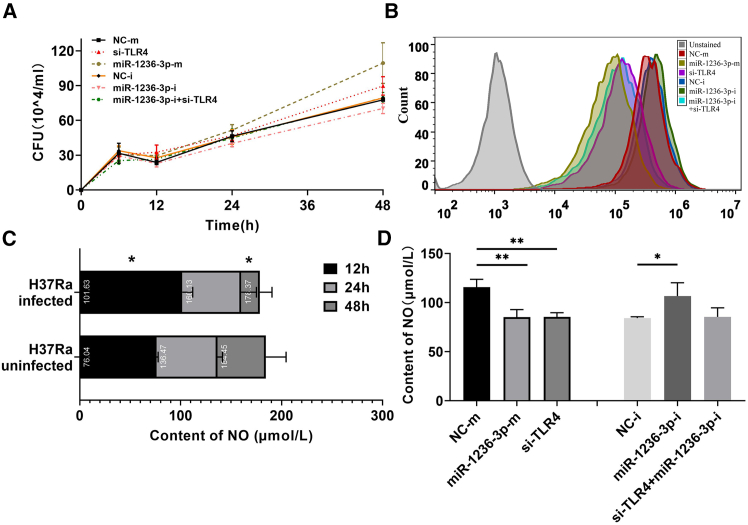
miR-1236-3p affects H37Ra survival and downregulates the production of ROS and NO by targeting TLR4 THP-1 cells were transfected with miR-1236-3p mimic, inhibitor or TLR4-specific siRNA (si-TLR4), respectively. At 24 h post-transfection, cells were infected with H37Ra at 5.0 MOI. At indicated time points post-infection, cells were harvested for CFU assay (A). At 24 h post-infection, cells were harvested for ROS assay (B) and NO assay (C and D). The production of ROS was determined by flow cytometry. All experiments were performed in triplicate, and data were presented as mean ± SD. Student’s *t* test was used for two groups and one-way ANOVA was used for the comparison of more than two groups. ∗, *p* < 0.05; ∗∗, *p* < 0.01; ∗∗∗, *p* < 0.001.

To investigate the impact of miR-1236-3p on the bactericidal capability of macrophages, the intracellular survival of *Mtb* was examined using CFU assay (Figures 4A and S1). The results showed that the number of intracellular viable bacteria decreased at 12 h and then gradually increased thereafter. Compared with the negative control group, cells treated with miR-1236-3p mimics and si-TLR4 showed significantly higher H37Ra survival rate, while the live bacterial count in cells treated with miR-1236-3p inhibitors was much lower, suggesting that miR-1236-3p may promote immune escape of *Mtb* by downregulating TLR4 (Figure 4A).

TLR4 signaling regulates the production of ROS and NO.^16^^,^^17^ ROS can execute bactericidal activity by oxidative destruction of membrane lipids, DNA, thiol, and tyrosine residues, whereas NO can exert toxicity by directly targeting the iron-sulfur cluster of central metabolic enzymes.^18^^,^^19^^,^^20^ We examined the effects of miR-1236-3p on the generation of ROS (Figure 4B) and NO (Figure 4C). THP-1-derived macrophages were transfected with miR-1236-3p-m, miR-1236-3p-i, or si-TLR4. The negative control for miRNA mimic (NC-m) or miRNA inhibitor (NC-i) were also included in the experiments. At 24 h post-transfection, cells were infected with H37Ra at 5.0 MOI and then ROS assay or NO assay were performed. The production of ROS (Figure 4B) was inhibited by miR-1236-3p mimic or TLR4-specific siRNA during *Mtb* infection. The NO production was inhibited by *Mtb* infection (Figure 4C) or miR-1236-3p mimic and TLR4-specific siRNA (Figure 4D) during *Mtb* infection, both of which could downregulate the expression of TLR4. However, these effects were restrained by miR-1236-3p inhibitor. Overall, the results suggest that *Mtb*-induced increase of miR-1236-3p may downregulate the production of ROS and NO by targeting TLR4 to facilitate the survival of *Mtb*.

### miR-1236-3p suppresses *Mtb*-induced NF-κB signaling activation in macrophages by targeting TLR4

**Figure 5:**
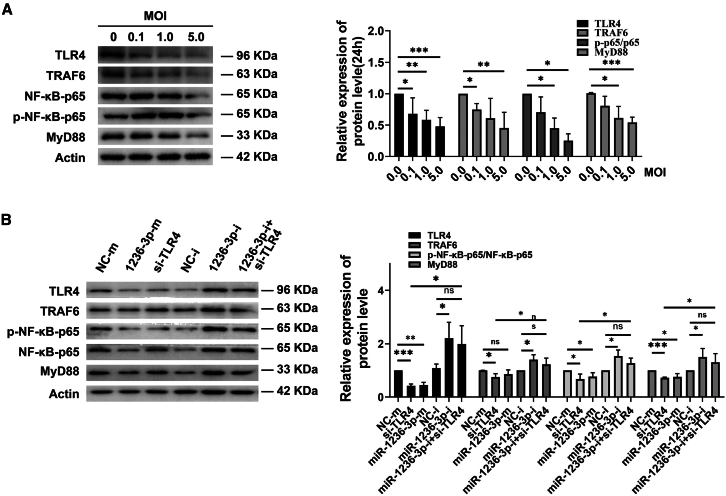
The effects of miR-1236-3p on NF-κB signaling pathway (A) The expression levels of TLR4, TRAF6, MyD88, NF-κB-p65, and p-NF-κB-p65 protein detected by Western blot assays in THP-1-derived macrophages infected with H37Ra at 5.0 MOI for 24h. (B) The protein levels of TLR4, TRAF6, and MyD88, in 0.5 MOI H37Ra-infected THP-1-derived macrophages transfected with si-TLR4 and miR-1236-3p mimic or inhibitor at 24h post-infection. All experiments were performed in triplicate and data are presented as mean ± SD. Student’s *t* test was used for two groups and one-way ANOVA was used for the comparison of more than two groups. ∗, *p* < 0.05; ∗∗, *p* < 0.01; ∗∗∗, *p* < 0.001.

TLR4 is required for NF-κB signaling pathway to induce innate immunity and subsequent inflammatory response. NF-κB, a critical element of innate immunity, serves as a multifunctional transcription factor that orchestrates the downstream expression of inflammatory and chemotactic factors, playing a pivotal role in cellular survival, immune responses, and inflammation.^21^ In MyD88-dependent pathway, the phosphorylation and subsequent degradation of IκB liberate NF-κB, allowing it to translocate into the nucleus and activate the production of inflammatory cytokines such as TNF-α, IL-1, and IL-6. As miR-1236-3p targets TLR4, we examined the role of miR-1236-3p in the activation of NF-kB signaling pathway during *Mtb* infection. First, THP-1-derived macrophages were infected with H37Ra at 0.1, 1.0, and 5.0 MOI and then the protein expression was analyzed at 24 h post-infection. The results showed that high level-/long time-H37Ra infection downregulated the phosphorylation level of NF-κB as well as the expression of TLR4, TRAF6, and MyD88 (Figures 5A and S2). Correspondingly, we detected the mRNA levels of key proteins in the TLR4-NF-κB pathway at 6h, 12h, and 24h. The results showed that long time H37Ra infection downregulated the mRNA levels of TLR4, TRAF6, MyD88, TNF-α, IL-6, and IL-1β (Figure S3). Next, THP-1-derived macrophages were transfected with si-TLR4, miR-1236-3p-m, miR-1236-3p-i, or NC-m, NC-i, and then infected with H37Ra at 5.0 MOI. At 24h post-infection, miR-1236-3p-m and si-TLR4 significantly reduced the expression of TLR4 and downregulated the phosphorylation level of p65, as well as the expression of TRAF6 and MyD88 (Figure 5B). Notably, the miR-1236-3p inhibitor significantly upregulated this response. Collectively, these data indicated that miR-1236-3p may downregulate NF-κB signaling and suppresses the inflammatory response through targeting TLR4.

## Discussion

**Figure 6:**
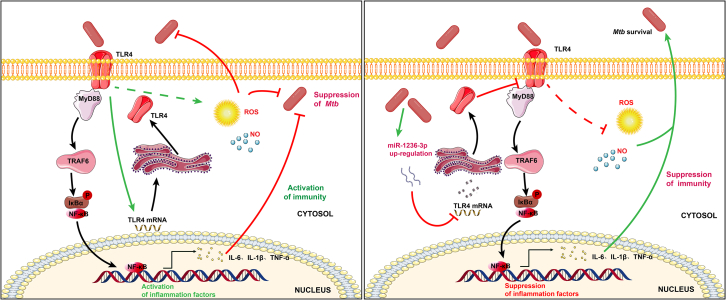
Diagram illustrating the role of miR-1236-3p in modulating the functions of *Mtb*-infected macrophages Left: *Mtb* recognition by TLR4 triggers the NF-κB signaling pathway, which promotes macrophage-mediated immune killing, effectively suppressing *Mtb* survival during initial infection. Right: miR-1236-3p targets TLR4 to downregulate the TLR4-mediated downstream NF-κB signaling pathway and reduce the production of antibacterial factors including NO and ROS, facilitating the survival of *Mtb* in Macrophages.

microRNAs have been reported to play multiple roles in reprogramming host’s transcriptome to adjust metabolism in response to *Mtb* infection.^22^ In the current study, we found that *Mtb* infection markedly enhanced the expression of miR-1236-3p in both THP-1 cells and clinical samples (Figure 1). The miR-1236-3p may downregulate the expression of TLR4 in macrophages by targeting the “G” allele located in the 3′-UTR of TLR4 polymorphism c.1205G>C (rs11536889), further leading to the decreased expression levels of MyD88 and TRAF6, the inhibition of NF-κB phosphorylation, and reduced production and release of ROS and NO (Figure 6). miR-1236-3p has also been reported as one of the factors affecting the risk at ventilator-associated pneumonia^23^ and periodontitis^24^ by binding to the “G” allele of TLR4 rs11536889. Our finding reveals a novel role for miR-1236-3p in modulating TLR-mediated pathways in *Mtb*-infected macrophages.

TLR4 plays critical roles in the interactions of *Mtb* with macrophages. TLR4 can recognize a number of *Mtb* antigens to induce different host immune responses against *Mtb*, including both innate immune defense and subsequent adaptive immune responses. *Mtb* infection of macrophages could induce cell death through apoptosis and necrosis. TLR4 was essential for balancing between necrotic and apoptotic cell death as necrosis was favored in the absence of TLR4 signaling in *Mtb* H37Rv-infected macrophages.^25^ *Mtb* proteins PE9 (Rv1088) and PE10 (Rv1089) can physically form a protein complex to bind TLR4, which increased the phosphorylation of IRF-3, modulated the expression of INF-β, IL-1b, and IL-10, and facilitated the apoptosis in THP-1 macrophages.^26^ However, the role of TLR4 in *Mtb*-infected animal models is still controversial. Aerosol *Mtb* challenge of mice defective in CD14, TLR2, or TLR4 revealed that TLR2-defective mice, rather than TLR4-defective one, is more susceptible to the high dose *Mtb*,^27^ suggesting that TLR-2 is an important *Mtb* resistance protein. In contrast, some studies show that TLR4 may play a key role in host resistance to *Mtb* infection *in vivo.*^28^^,^^29^ TLR4 mutant C3H/HeJ mice have reduced capacity to eliminate mycobacteria from the lungs and increased spreading of infection to spleen and liver, with 10–100 times higher bacteria burden levels than the wild-type mice.^28^^,^^29^ In addition, the lungs of TLR4 mutant mice showed chronic pneumonia characterized by strong neutrophil infiltration, reduced macrophages recruitment, and abundant acid-fast bacilli. Moreover, C3H/HeJ mice produced lower level of pulmonary expression of TNF-α, IL-12p40, and monocyte chemoattractant protein 1.^28^^,^^29^ Those results demonstrate that impaired TLR4 may result in chronic infection with compromised capability to eliminate mycobacteria. This phenomenon is consistent with the observation in our report that ATB and LTBI patients had the lower expression of TLR4 in peripheral blood (Figure 3), which may be causally associated with the increased expression of miR-1236-3p in clinical *Mtb* infection or *in vitro* cell model experiments.

Inhibition of TLR4 could reduce phagocytosis and suppress the TLR4-dependent proinflammatory response,^30^ which are all closely associated with the elimination of *Mtb* in macrophages. THP1-derived macrophages express higher surface TLR4 and NOX2 following LPS treatment. NOX2, a prototypical member of the NADPH oxidase NOX superfamily, is responsible for the generation of ROS in innate and adaptive immunity.^31^ However, both TLR4 and NOX2 were reduced by *Mtb* infection,^30^ indicating that the *Mtb* infection downregulates the role of TLR4-NOX2 axis in macrophage activation. More evidences show that TLR4 plays a critical role in mediating ROS and NO production.^32^^,^^33^^,^^34^ In addition, the activation of the canonical NF-κB signaling is essential to the inflammatory response in controlling innate immunity.^35^ In our study, the over-expressed miR-1236-3p attenuated inflammatory response by inhibiting the phosphorylation of NF-κB and the expression levels of MyD88 and TRAF6 and other proteins in TLR4/MyD88/NF-κB pathway. In agreement with this observation, we found that miR-1236-3p also reduced the production of ROS and NO in accompany with the downregulated TLR4 (Figure 6). Thus, miR-1236-3p could be a key factor in regulation of TLR4 signaling in macrophages during *Mtb* infection.

The interaction of invading pathogenic bacteria with macrophages ignites the inflammatory response to produce a variety of pro-inflammatory cytokines such as TNF-α, IL-1β, IL-6 until the pathogens are eliminated and/or anti-inflammatory regulatory mechanisms take over.^36^ The phagocytosis stimulates macrophages to produce a respiratory burst and generate reactive oxygen and nitrogen intermediates to limit the growth of intracellular bacteria including *Mtb*.^37^ However, *Mtb* is able to resist inflammatory cytokines, by establishing a niche for survival in macrophages^30^^,^^38^ and even hijack host innate immune pathways for their own benefit.^39^

In summary, the results in this report revealed a mechanism by which *Mtb* upregulates miR-1236-3p expression to reduce the immune capacity of macrophages and even ultimately contribute to a poor clinical outcome. Our findings are valuable for clinic utility, for instance, elevated miR-1236-3p in peripheral blood may serve as a diagnostic biomarker to differentiate ATB/LTBI from HC, while therapeutic strategies targeting miR-1236-3p silencing or TLR4/NF-κB pathway activation could restore macrophage bactericidal activity, and the rs11536889 SNP in TLR4 may enable personalized interventions based on genetic profiles.

### Limitations of the study

Several limitations in this study should be acknowledged. Although we excluded the patients with other chronic respiratory diseases and matched the age and gender to reduce the inconsistency of this study, other factors such as the variablity of the timepoints of blood samplings, limited subject sample size, and the ethnic bias may also be involved in the results. Further study of expanded population is required to provide more evidence related with our findings, particularly the correlation of TLT4 level with the rs11536889 SNP in individuals, to substantiate that the occurrence of SNP results in the loss of the target site for miR-1236-3p, leading to a restoration of TLR4 levels. This modulation is beneficial for the host’s immune response against *Mtb*. In addition, the mechanism by which *Mtb* causes upregulation of miR-1236-3p expression has yet to be explored. One study reported that IL-1β can affect the level of miR-1236-3p.^40^ But the cellular pathways controlling miR-1236-3p in tuberculosis are still unknown. It is also possible that miR-1236-3p may target other genes during *Mtb* infection, such as MyD88, as confirmed in Ulcerative colitis but not in TB.^41^

In addition, *Mtb* strain H37Ra was used in this study. H37Ra is an avirulent strain attenuated from the virulent strain H37Rv of *Mtb*, which basically retains the immunogenicity of the virulent strain and has similar immunogenicity with the H37Rv virulent strain, causing similar immune processes.^42^ The Beijing strain *Mtb* has garnered significant clinical attention due to its widespread presence, especially in East Asia, and its link to multidrug-resistant tuberculosis.^42^ Thus, it would be an interesting work to explore the role of miRNAs identified in this study in the context of Beijing stain-mediated infection in future research.

## Resource availability

### Lead contact

Further information and requests for resources and reagents should be directed to and will be fulfilled by the lead contact, Li Dong (dongli@sxu.edu.cn).

### Materials availability

This study did not generate unique reagents.

### Data and code availability

All data reported in this paper will be shared by the lead contact upon request.

This paper does not report original code.

Any additional information required to reanalyze the data reported in this paper is available from the lead contact upon request.

## Acknowledgments

We are grateful to everyone who provided blood samples and consents for genetic analysis and to Drs. Hongli Bu and Quanhong Wang for blood collection, Drs. Guangxin Chen and Jiangdong Wu for data analysis, and all other clinicians and coordinators for their contributions to the work.

This work was supported by the Key Research and Development Program of Shanxi province (grant number: 202102130501009); Central Guidance Fund for Local Scientific and Technological Development (grant number: YDZJSXD2024008); Four “batches” innovation project of invigorating medical through science and technology of Shanxi province (2023XM015), Science and Technology Plan Project of Hulunbuir (Grant No. SF2023015).

## Author contributions

L.D., H.D., and J.H. conceived and designed the experiments. J.H., Q.Y., Q.L., and T.W. collected the experimental specimens and clinical data. Y.T.Z., J.H., and X.C. performed the experiments. Y.T.Z., J.H., and X.C. performed the data analysis. L.D. and C.W. provided administration. Y.T.Z., J.H., L.D., and L.X. wrote and revised the manuscript. All authors contributed to the article and approved the submitted version.

## Declaration of interests

The authors declare that the research was conducted in the absence of any commercial or financial relationships that could be construed as a potential conflict of interest.

## STAR★Methods

### Key resources table

**Table:** 

REAGENT or RESOURCE	SOURCE	IDENTIFIER
**Antibodies**		

anti-p-p65	Santa Cruz Biotechnology	sc-136548
anti-p65	Proteintech	66535-1-Ig
anti-β-actin	Boster	BA2305
anti-TLR4	ABclonal	A0007
anti-TRAF6	ABclonal	A16991
anti-MyD88	ABclonal	A0980
goat anti-rabbit IgG	Proteintech	SA00001-2
goat anti-mouse IgG	ABclonal	AS003

**Bacterial and virus strains**		

tdTomato-H37Ra	Institutes of Biomedical Sciences Shanxi University	N/A

**Biological samples**		

peripheral blood of study population	Taiyuan Fourth People’s Hospital of China	N/A

**Chemicals, peptides, and recombinant proteins**		

phorbol-12-myristate acetate (PMA)	Sigma	P8139
2,7′-dichlorodihydrofluorescein diacetates (DCFDA)	Beyotime	S0033M

**Experimental models: Cell lines**		

Human acute monocyte leukemia cell line THP-1	National Infrastructure of Cell Line Resource (Beijing)	4201HUM-CCTCC00100
human renal embryonic cell	National Infrastructure of Cell Line Resource (Beijing)	1101HUM-PUMC000091

**Oligonucleotides**		

Si-TLR4 Sense:5′-GCCAGUUUCUGGCAUAUUATT-3′ Antisense:5′-UAAUAUGCCAGAAACUGGCTT-3′	This paper	N/A
NC mimic Sense:5′-UUCUCCGAACGUGUCACGUTT-3′ Antisense:5′-ACGUGACACGUUCGGAGAATT-3′	This paper	N/A
NC inhibitor Sense:5′-CAGUACUUUUGUGUAGUACAA-3′	This paper	N/A
miR-1236-3p mimic Sense: 5′-CCUCUUCCCCUUGUCUCUCCAG-3′ Antisense:5′-GGAGAGACAAGGGGAAGAGGUU -3′	This paper	N/A
miR-1236-3p inhibitor Sense:5′-CUGGAGAGACAAGGGGAAGAGG-3′	This paper	N/A
β-actin Primer sequences F:AGTGTGACGTGGACATCCGCA R: ATCCACATCTGCTGGAAGGTGGAC	This paper	N/A
TLR4 Primer sequences F:GACTGGGTAAGGAATGAGCTAG R: ACCTTTCGGCTTTTATGGAAAC	This paper	N/A
TRAF6 Primer sequences F:GAGACAGGTTTCTTGTGACAAC R: TGGCAACCAAAAGTACTGAATG	This paper	N/A
MyD88 Primer sequences F:AATCTTGGTTCTGGACTCGCCTTG R: AGCACAGATTCCTCCTACAACGAAAG	This paper	N/A
TNF-α Primer sequences F:AAGGACACCATGAGCACTGAAAGC R: AGGAAGGAGAAGAGGCTGAGGAAC	This paper	N/A
IL-1β Primer sequences F:CCACAGACCTTCCAGGAGAATG R: GTGCAGTTCAGTGATCGTACAGG	This paper	N/A
IL-6 Primer sequences F:AGACAGCCACTCACCTCTTCAG R: TTCTGCCAGTGCCTCTTTGCTG	This paper	N/A
miR-1236-3p RT Primer sequences TCGTATCCAGTGCAGGGTCCGAGGTATTCGCAC TGGATACGACCTGGAG	This paper	N/A
miR-1236-3p Primer sequences F:GCGCCTCTTCCCCTTGTCT R: AGTGCAGGGTCCGAGGTATT	This paper	N/A

**Recombinant DNA**		

psiCHECK™-2 Vectors	Promega	C8021

**Software and algorithms**		

EpiData 3.1	EpiData Association, Denmark	https://epidata.dk/download.php
SPSS version 20.0	SPSS Inc., Illinois, USA	https://www.ibm.com/support/pages/downloading-ibm-spss-statistics-20
TargetScan Release 8.0 platform	Whitehead Institute for Biomedical Research	https://www.targetscan.org/vert_80/

### Experimental model and study participant details

#### Human samples

A total of 81 subjects were recruited from the Chinese Han population, including 27 ATB, 28 LTBI and 26 HC. ATB patients were diagnosed by clinicians in Taiyuan Fourth People’s Hospital of China. HC and LTBI subjects were also recruited in the same hospital and group-matched by mean age or gender.

The criteria for ATB were defined by the national guidelines for Diagnosis of Pulmonary Tuberculosis (WS288-2017) and Classification of Tuberculosis (WS196-2017). The LTBI population was defined as individuals who was tested positive using Interferon Gamma Release Assay (IGRA), but without clinical symptoms (e.g., cough, fever, weight loss) and abnormal chest X-ray findings. ATB patients with history of anti-TB drugs treatment were excluded. We also excluded ATB or LTBI patient who has comorbidities such as cancers, diabetes, immune system diseases, or chronic respiratory diseases. All protocols were approved by the Medical Ethics Committee of Taiyuan Fourth People’s Hospital (No. 2019-008) and the informed consent was signed by all subjects. Detailed information about the participants is provided in Table 1.

#### Cells

Human acute monocyte leukemia cell line THP-1 was obtained from National Infrastructure of Cell Line Resource (Beijing, China) and maintained in RPMI-1640 medium (Boster) supplemented with 10% fetal bovine serum (FBS, Gibco/Sigma, USA) and penicillin-streptomycin (100 g/mL) at 37°C in a humidified atmosphere with 5% CO_2_. The THP-1 was tested negative for mycoplasma.

Cells were seeded at a density of 2 × 10^5^ cells/mL in 6-well pre-coated plates for enhanced adherence. THP-1 was differentiated into macrophages by treatment with 100 nM phorbol-12-myristate acetate (PMA, Sigma, USA) for 24 h.

### Method details

#### *Mtb* culture and infection of THP-1 macrophages

The recombinant *Mtb* strain pTEC27-tdTomato was generated through plasmid transformation of the parental H37Ra. This strain carries the pTEC27-tdTomato plasmid (conferring hygromycin resistance and expressing the fluorescent reporter tdTomato under a constitutive mycobacterial promoter), which was generously provided by Prof. Lalita Ramakrishnan at the Department of Medicine, University of Cambridge, UK. The strain is preserved in the Institutes of Biomedical Sciences, Shanxi University. *Mtb* strain was grown at 37°C in Middlebrook 7H9 broth medium supplemented with 10% BD Middlebrook ADC, 0.05% Tween 80, and 100 ng/mL Hygromycin B (Solarbio, China) with continuous agitation (220 rpm) until mid-log phase growth.

THP-1-derived macrophage was infected with *Mtb* at 37°C at 0.1, 1.0, or 5.0 MOI, respectively. Extracellular bacteria were removed by three washes with warm PBS, and then the fresh medium containing 2 μg/mL gentamicin (Solarbio) was added to suppress residual extracellular growth of bacteria.

#### Small RNA transfection

THP-1 cells were transfected with different small RNAs including miR-1236-3p mimic (miR-1236-3p-m), miR-1236-3p inhibitor (miR-1236-3p-i), TLR4-specific siRNA (si-TLR4), mimic NC, and NC inhibitor (purchased from the Shanghai GenePharma Company, China) using lipofectamine 2000 (Thermo Fisher, USA). The sequences of those small RNAs are listed in Table S1.

#### Real-time PCR

Total cellular RNA was extracted using TRIzol (Invitrogen). MiRNA and total RNA in clinical samples were extracted using miRcute miRNA extraction kit (Tiangen, China) according to the manufacturer’s instruction. cDNA was synthesized using the PrimeScript RT reagent kit (Takara). Real-time PCR was performed in triplicate using CFX Connect™ Real-Time System (Bio-Red, USA). For miRNA quantification, STEM-LOOP RT-qPCR was used. The relative levels of miRNA and mRNA were determined by normalizing to the transcripts of U6 (Bulge-Loop U6 qPCR Primer Set, RIBOBIO) and β-actin, respectively, using ΔΔCt method. The primer sequences were shown in Table S2.

#### Dual-luciferase assay

The psiCHECK™-2 dual-luciferase reporter vector (Promega) was utilized to evaluate miRNA-1236-3p binding to the TLR4 3′-untranslated region (3′-UTR). THP-1 cells were co-transfected with luciferase reporter plasmids containing wild-type or mutant 3′-UTR of TLR4 and different small RNAs including miR-1236-3p mimic, NC mimic, miR-1236-3p inhibitor, or NC inhibitor. Luciferase activity was determined at 48 h post-transfection according to the manufacturer’s protocol.

#### Biotinylated miRNA Pull Down Assay

The biotin 3′-labeled miRNA (Bio-miRNA) was synthesized and purchased from Shanghai GenePharma Company. 100nM of bio-miRNA were transfected into 293T cells using lipofectamine 2000 (Thermo Fisher). Forty-eight hours post-transfection, whole cell lysates were prepared. RNA isolation, pulldown, and RT-qPCR procedure were processed as per the instructions of BersinBio™ miRNA pulldown Kit (BersinBio™, China).

#### Western blot analysis

Cell lysates were prepared using 2% SDS lysis buffer, and proteins were analyzed by Western blotting. β-actin serves as a loading control. The primary antibodies used in this study include anti-p-p65 (sc-136548, Santa Cruz Biotechnology), anti-p65 (66535-1-Ig, Proteintech), anti-β-actin (BA2305, Boster), anti-TLR4 (A0007, ABclonal), anti-TRAF6 (A16991, ABclonal), and anti-MyD88 (A0980, ABclonal). Secondary antibodies used in Western blot analysis include goat anti-rabbit IgG (H + L, SA00001-2, Proteintech) and goat anti-mouse IgG (H + L, AS003, ABclonal).

#### Colony forming unit (CFU) assay

THP-1 cells were infected with *Mtb* at 0.1, 1.0, or 5.0 MOI. After 4 h of incubation, the infected cells were washed 3 times with PBS. The cells were treated with gentamicin for 2 h to remove extracellular bacteria and then lysed with 0.1% Triton X-100 at the indicated time points. Homogenates underwent 10-fold-serial dilution and each dilution was inoculated on 7H10 agar plates supplemented with 10% OADC. Plates were incubated at 37°C for 3 weeks before the colonies were counted manually.

#### NO assay

THP-1-derived macrophages were infected with *Mtb* as described in previous sections. Supernatants were collected at 24-h intervals, centrifuged at 12,000 × *g* for 10 min at 4°C to remove cellular debris, and aliquoted into RNase/DNase-free microtubes (Axygen). Samples were stored at −80°C until analysis (within 2 weeks to prevent nitrite degradation). The concentration of NO was measured using Nitric Oxide assay kit (Nanjing Jiancheng Bioengineering Institute) and Agilent Technologies Cary 60 UV-Vis ultraviolet spectrophotometer at 550 nm.

#### Measurement of intracellular reactive oxygen species (ROS)

Intracellular ROS levels were measured using a ROS indicator, 2,7′-dichlorodihydrofluorescein diacetates (DCFDA, S0033M, Beyotime, China), at a final concentration of 10 μM. Cells were incubated for 20 min in the dark, then collected and analyzed using CytoFLEX LX flow cytometer (BECKMAN).

### Quantification and statistical analysis

Dataset in EpiData 3.1 (EpiData Association, Denmark) was analyzed with SPSS version 20.0 (SPSS Inc., Illinois, USA). The differences in the distributions of demographic characteristics between TB and healthy control groups were compared by the independent two-sample *t*-test for continuous variables or by the χ2-test for categorical variables. Triple tests were conducted for each experiment. Statistical significance analysis was performed using Student’s *t*-test between two groups and one-way ANOVA among multiple groups.

### Additional resources

The work is not part of/involves a clinical trial.
