# Supplementary material for: miR-1236-3p targets Toll-like receptor 4 to suppress the anti-Mycobacterium tuberculosis activity of macrophage
Source: iScience. 2025 May 8;28(6):112522. doi: 10.1016/j.isci.2025.112522 (PMC12152351; doi:10.1016/j.isci.2025.112522)
Supplement: Document S1. Figures S1–S3 [file mmc1.pdf]

iScience, Volume *28*

Supplemental information

miR-1236-3p targets Toll-like receptor 4 to suppress the anti-*Mycobacterium tuberculosis* activity of macrophage

Yating Zhang, Jie Han, Qianwei Yang, Xiaogang Cui, Huiping Duan, Ting Wu, Changxin Wu, Li Xing, Qunqun Liu, and Li Dong

Supplementary Figures:

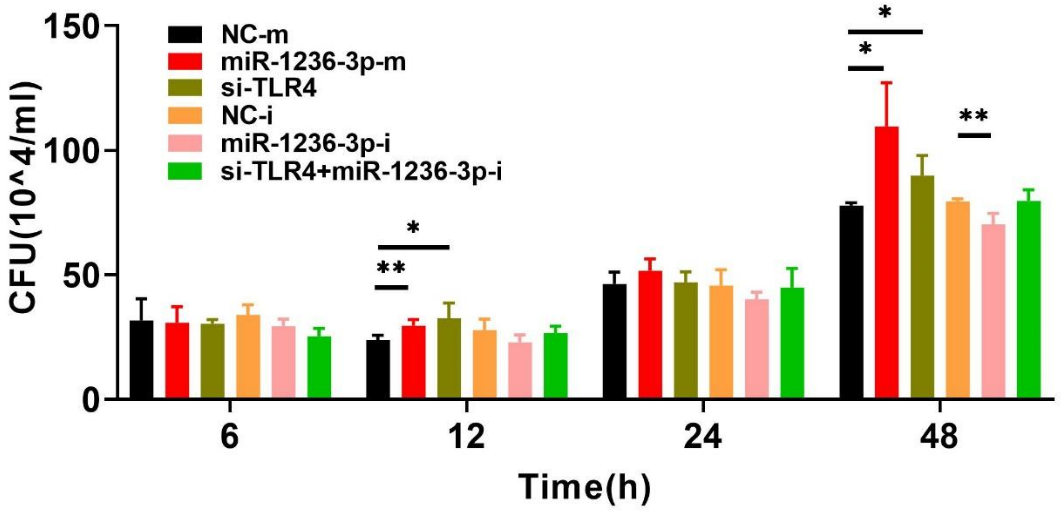

Supplementary figure 1. THP-1 cells were transfected with miR-1236-3p mimic, inhibitor or siRNA targeted TLR4. 24h after transfection, cells were infected with H37Ra at an MOI of 5.0. 24h post-infection, cells were harvested for CFU assay. This figure serves as a supplement to Fig.4A.

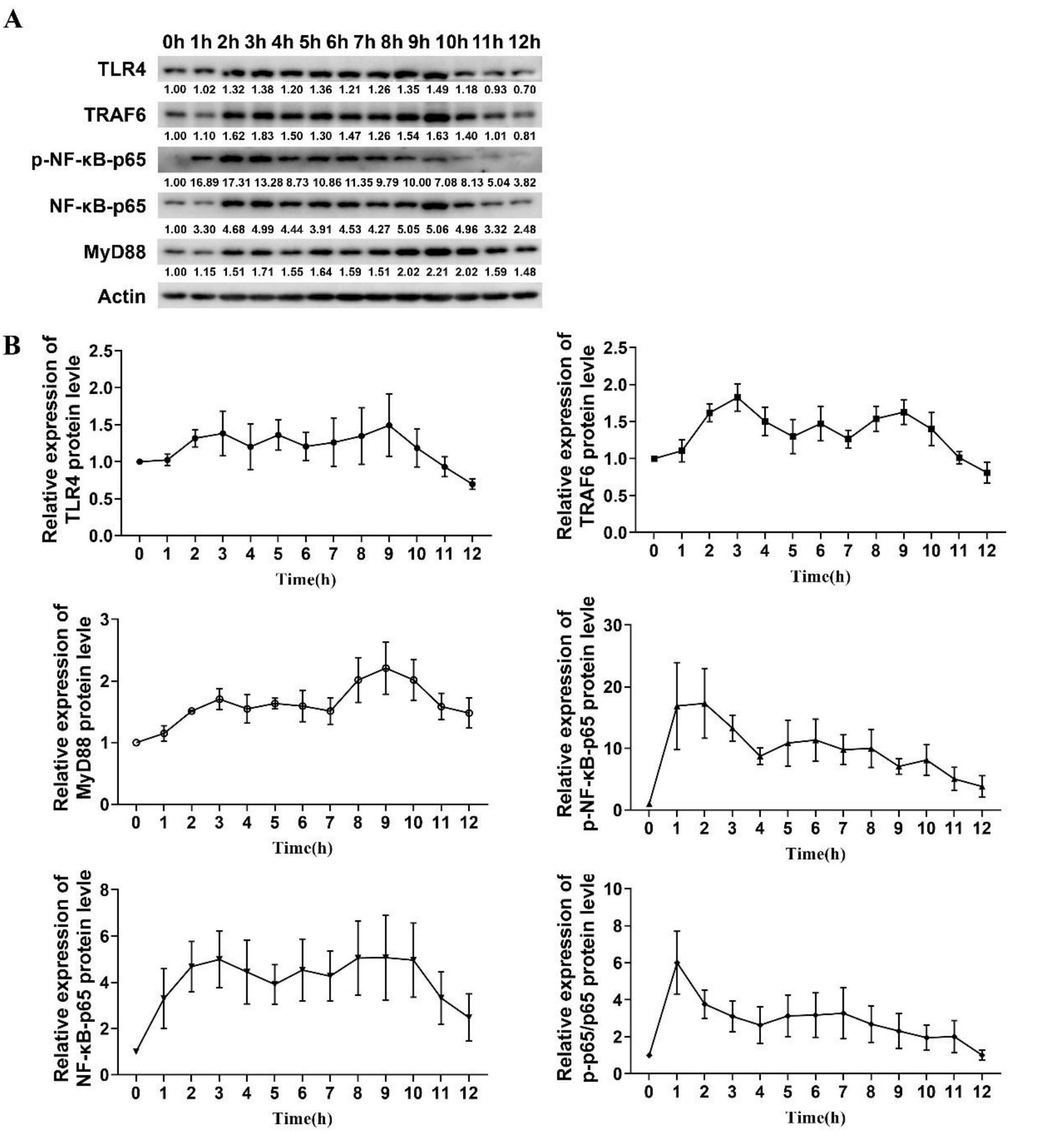

Supplementary figure 2. The expression levels of TLR4, TRAF6, MyD88, p-NF-κB-p65, NF-κB-p65 protein and the phosphorylation level of p65 were analyzed using western blot assays in THP-1 cells with H37Ra infection (MOI=5.0, Time= 0h to 12h).

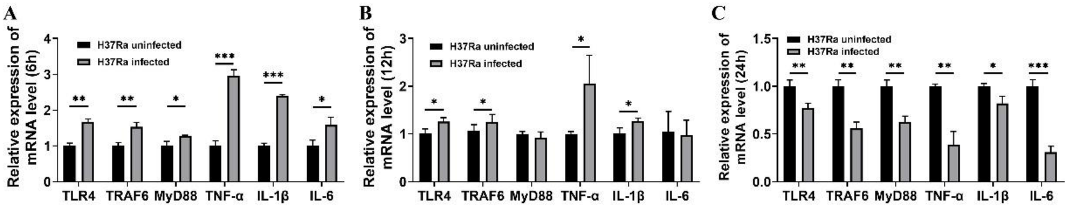

Supplementary figure 3. The mRNA levels of TLR4, TRAF6, MyD88, TNF-α, IL-6 and IL-1β of THP-1 cells with H37Ra infection (MOI=5.0), detected by qRT-PCR at 6h (A), 12h (B) and 24h (C).
