# Supplementary material for: miR-1236-3p targets Toll-like receptor 4 to suppress the anti-Mycobacterium tuberculosis activity of macrophage
Source: iScience. 2025 May 8;28(6):112522. doi: 10.1016/j.isci.2025.112522 (PMC12152351; doi:10.1016/j.isci.2025.112522)
Supplement: Document S2. Tables S1 and S2 [file mmc2.pdf]

Supplementary Table S1. RNA oligo used in transfection

Gene	sequences
Si-TLR4	Sense:5’-GCCAGUUUCUGGCAUAUUATT-3’
	Antisense:5’-UAAUAUGCCAGAAACUGGCTT-3’
NC mimic	Sense:5’-UUCUCCGAACGUGUCACGUTT-3’
	Antisense:5’-ACGUGACACGUUCGGAGAATT-3’
NC inhibitor	Sense:5’-CAGUACUUUUGUGUAGUACAA-3’
miR-1236-3p mimic	Sense:5’-CCUCUUCCCCUUGUCUCUCCAG-3’
	Antisense:5’-GGAGAGACAAGGGGAAGAGGUU -3’
miR-1236-3p inhibitor	Sense:5’-CUGGAGAGACAAGGGGAAGAGG-3’

Supplementary Table S2. Primer sequences for RT-qPCR

Gene		Sequences (5'-3')
β-actin	Forward	AGTGTGACGTGGACATCCGCA
	Reverse	ATCCACATCTGCTGGAAGGTGGAC
TLR4	Forward	GACTGGGTAAGGAATGAGCTAG
	Reverse	ACCTTTCGGCTTTTATGGAAAC
TRAF6	Forward	GAGACAGGTTTCTTGTGACAAC
	Reverse	TGGCAACCAAAAGTACTGAATG
MyD88	Forward	AATCTTGGTTCTGGACTCGCCTTG
	Reverse	AGCACAGATTCCTCCTACAACGAAAG
TNF-α	Forward	AAGGACACCATGAGCACTGAAAGC
	Reverse	AGGAAGGAGAAGAGGCTGAGGAAC
IL-1β	Forward	CCACAGACCTTCCAGGAGAATG
	Reverse	GTGCAGTTCAGTGATCGTACAGG
IL-6	Forward	AGACAGCCACTCACCTCTTCAG
	Reverse	TTCTGCCAGTGCCTCTTTGCTG
miR-1236-3p	RT primer	GTCGTATCCAGTGCAGGGTCCGAGG
		TATTCGCACTGGATACGACCTGGAG
	Forward	GCGCCTCTTCCCCTTGTCT
	Reverse	AGTGCAGGGTCCGAGGTATT
